# Revealing Synergistic Mechanism of Multiple Components in Gandi Capsule for Diabetic Nephropathy Therapeutics by Network Pharmacology

**DOI:** 10.1155/2018/6503126

**Published:** 2018-04-26

**Authors:** Jian Zhang, Qiqiang Zhang, Xiaofei Chen, Yan Liu, Jiyang Xue, Arik Dahan, Hai Zhang, Yifeng Chai

**Affiliations:** ^1^Department of Pharmacy, Xinhua Hospital, Shanghai Jiao Tong University School of Medicine, Shanghai 200092, China; ^2^School of Pharmacy, Second Military Medical University, Shanghai 200433, China; ^3^Department of Pharmacy, Shanghai First Maternity and Infant Hospital, Tongji University School of Medicine, Shanghai 201204, China; ^4^Department of Clinical Biochemistry and Pharmacology, School of Pharmacy, Ben-Gurion University of the Negev, 84105 Beer-Sheva, Israel

## Abstract

Gandi capsule, a traditional Chinese herbal medicinal formulation that consists of eight herbs, is used as a clinical therapy for diabetic nephropathy. To clarify the potential synergistic mechanism, this study adopted a network pharmacology strategy to screen the action targets that corresponded to the active components in the Gandi capsule. We first constructed a compound database of 315 components in the Gandi capsule and a target database of diabetic nephropathy, which included 155 target proteins. Six representative compounds were selected to dock with 99 proteins found in the UniProtKB database with their PDB code, and interaction networks between the active ingredients of the Gandi capsule and their targets were mapped out. Results revealed 47 proteins with a high affinity with at least one compound molecule in the Gandi capsule. The main action pathways closely related to the development of diabetic nephropathy were the TGF-*β*1, AMPK, insulin, TNF-*α*, and lipid metabolism pathways as per network pharmacology analysis. In the interaction network, ACC1, SOD2, COX2, PKC-B, IR, and ROCK1 proteins had the most frequent interactions with the six compounds. We performed visual molecular docking in silico and experimentally confirmed competitive component-protein binding by SPR and an enzyme activity test, which highlighted the relationships of wogonin to COX2 and SOD2, astragaloside IV to ACC1, and morroniside to ACC1. We concluded that the potential synergistic mechanism of the Gandi capsule resulted from high affinities with multiple proteins and intervention in multiple pathways in combination therapy of diabetic nephropathy.

## 1. Introduction

Diabetic nephropathy is a severe complication of diabetes mellitus, especially type 2 diabetes. One-third of patients with type 2 diabetes will develop diabetic nephropathy, which ultimately leads to end-stage renal disease (ESRD) [1]. Moreover, diabetic nephropathy is also the major cause of ESRD in China and behind glomerulonephritis. After diabetic nephropathy has developed into ESRD, kidney failure is unavoidable and irreversible and can only be treated with renal replacement [2]. Meanwhile, although some therapeutic methods can relieve the symptoms of diabetic nephropathy, including strict control of blood pressure, the administration of angiotensin-converting enzyme inhibitors, and the pharmacological suppression of the renin-angiotensin system, there are few methods currently available to reverse the development of diabetic nephropathy [3].

Gandi capsule (GDC) is a traditional Chinese medicinal formulation created by the Pharmacy Department of Xinhua Hospital, Shanghai Jiao Tong University, and used for diabetic nephropathy. It consists mainly of eight traditional Chinese herbs:* Astragali Radix, Corni Fructus, Radix Rehmanniae, Leonuri Herba, Sophorae Flos, Scutellariae Radix*,* Bombyx Batryticatus*, and* Phyllanthi Fruit* [4]. Thus, it is clear that GDC is a broad-spectrum formulation with many compounds. Many herbs in GDC have been confirmed to have positive effects on diabetic nephropathy, such as Astragali Radix, a monarch medicine in GDC, which was demonstrated to reduce levels of fasting blood glucose and albuminuria and ameliorate the pathological changes of early diabetic nephropathy in animals [5]. Moreover, many components from GDC have been shown to have a positive influence in diabetic nephropathy. For example, some studies have shown that loganin exerts a renal protective role in diabetic nephropathy through the reduction of renal connective tissue growth factor (CTGF) expression through the extracellular-signal-regulated kinase (ERK) signaling pathway [6]. Moreover, calycosin and calycosin-7-O-*β*-D-glucoside inhibited high glucose-induced mesangial cell proliferation and AGE-induced endothelial cell apoptosis in diabetic nephropathy [7]. Meanwhile, some studies have indicated that quercetin may ameliorate the epithelial-mesenchymal transition in diabetic nephropathy through the inhibition of the two transcriptional factors (Snail and Twist) and the activation of mTORC1/p70S6K [8]. A previous study has described the characteristics of GDC from several perspectives, such as the analysis of ingredients in GDC [4]. However, it is still unclear if the synergistic mechanism of multiple components in the GDC acts on multiple targets for the therapy of diabetic nephropathy. It is known that the pharmacological mechanisms of traditional Chinese medicine (TCM) arise from the multiple compounds with multiple targets and synergistic interactions, so it is difficult to clarify the comprehensive pharmacological mechanisms [9].

To reveal the main pharmacological action mechanisms of GDC, we employed a network pharmacology strategy. Network pharmacology, which is based on the interaction network of diseases, genes, target proteins, and drugs, is a systematic analytical method [10]. This method emphasizes the translation from the opinion of one target to one drug to a novel strategy of multicomponent to multitarget [11]. It can reveal the action mechanism of the drug through the combination of computational biology, systems biology, and “omics” related to target drug [12]. Recent reports have emphasized the broad application of network pharmacology to determine the mechanism of action of TCM formulation. For example, the network pharmacology approach was used to define the active components and potential targets in* Curculigo orchioides* for the treatment of osteoporosis [13]. In this method, multiple target synchronous regulation can be verified, which supports the complexity of TCMs. In this study, two other methods, molecular docking and network analysis, were involved. Molecular docking is a common approach for the identification of the interactions among the receptor protein and molecules with high accuracy [14]. Therefore, we can screen for potential molecules with a high binding affinity. Network analysis is based on the features of the diagram drawn based on the interactions between the protein and protein or molecule.

Therefore, based on the advantages of network pharmacological research for the study of TCM, the validation of the binding affinity between the proteins and molecules was undertaken. Furthermore, special proteins with specific network pharmacological mechanisms in diabetic nephropathy can be identified. The results could provide a better understanding of the synergistic effect of compounds in GDC for the treatment of diabetic nephropathy. Moreover, the network pharmacology strategy could assist researchers in the simplification of the study of complex TCM formulas and provide a novel strategy to research the mechanism of action of other complex TCM formulae.

## 2. Materials and Methods

### 2.1. Materials

Standard substances, including astragaloside IV, morroniside, and wogonin, were purchased from Standard (Shanghai), acetyl-CoA carboxylase 1 was purchased from Proteintech (Shanghai), and cyclooxygenase-2 and superoxide dismutase were purchased from Sino Biological (Shanghai). Other kinase proteins were purchased from Proteintech (Shanghai).

### 2.2. Construction of Compound and Protein Target Database

We collected the compounds from the original herbs in GDC from the Chemistry Database developed by Shanghai Organic Institute. A proportion of the representative compounds were selected out to proceed with the docking. These compounds were imported into PubMed to find their structure, canonical SMILES, and any relevant other features. Meanwhile, we compiled the protein database about diabetes, diabetic complication, diabetic nephropathy, and microvascular disease collected from Therapeutic Target Database (http://bidd.nus.edu.sg/group/cjttd/) and DrugBank (http://www.drugbank.ca/). To identify the complete features of GDC, no prescreening filters were applied. If a PDB code was available in UniProtKB (http://www.uniprot.org/) of these proteins with lower resolution, their crystal structures were derived from the Research Collaboratory for Structural Bioinformatics (RCSB) Protein Data Bank (http://www.pdb.org/) with their own ligands. All protein structures used were from humans.

### 2.3. Molecular Docking

In this step, the canonical SMILES of the compounds selected were imported into Discovery Studio 3.0 to define the structures of these molecules and the structures of proteins were also entered. At first, the Preparation Module was used to clean the protein structure by using the CHARMM force field. The Preparation Module was used to optimize the structures so that they could be adapted to docking. Molecular docking was used to prove the potential interaction between the proteins and the drug molecules. The receptor-ligand interaction module of Discovery Studio 3.0 was used in our study at the earliest. Specific binding sites in the target protein were defined based on the crystal structure of the protein-ligand compounds and the docking procedure was used to dock the ligand into a specific binding site. To generate all possible conformations of molecules, a conformational search was applied. The generated conformations were matched with a binding site to select the optimal conformations, which could interact with the binding site. This generated a LibDock score if the molecule was successfully docked with the target proteins and the LibDock score of the ligand provided the baseline value to define the affinity between the protein and molecule. The whole docking procedure was conducted under the CHARMM force field. The docking tolerance was set to 0.25, the docking performance was set to be high quality, the conformation method was set to be FAST, the energy threshold was 20.0, and the minimization algorithm was set to smart minimizer. The default values were selected for other parameters.

### 2.4. Construction of Compound-Target Interaction Network

To analyze the interaction of the compound-protein-pathway in diabetic nephropathy, we used analytical software, Cytoscape 3.51, to construct a network diagram. The compound-protein network was founded by the interaction of the candidate molecule with the corresponding proteins. The protein-pathway network was built through the connection of the proteins and their own pathways, respectively. In the network, the nodes represent compounds, proteins, and pathways; the edges represent the interactions between the molecule, protein, and pathway. The information was collected from TTD (http://bidd.nus.edu.sg/group/cjttd/) and KEGG (http://www.genome.jp/kegg/pathway.html). The network parameters were calculated by the network analyzer module in Cytoscape 3.51. Only results with a high binding affinity in molecular docking could be used for network analysis.

### 2.5. SPR Assay

In order to prove the validity of the network pharmacological method, different protein-molecule interactions were selected to participate in this surface plasmon resonance (SPR) test. In the SPR test, the target proteins were first combined with the CM5 chip. After correction through standard solutions, a series of different concentrations of molecules (0, 2, 4, 8, 16, 32, 64, and 128 *μ*mol/l) were tested for their binding to the protein. Finally, the affinity between the protein and molecule was determined by an array of parameters, such as the *Kd* values,* R*max, offset, and *χ*^2^.

### 2.6. Enzyme Activity Test

As there were some kinase proteins in our protein database with different characteristics to other proteins, we used an enzyme activity test to validate the real interactions between the protein and molecule.

Selected pairs of protein and molecule participated in enzyme activity test, while electrophoretic mobility shirt assay (EMSA) and Lance ultra were conducted in an experiment with ten different concentrations (100, 50, 25, 12.5, 6.25, 3.12, 1.56, 0.78, and 0.39 *μ*mol/l). Finally, the IC_50_ (nM) value was used to assess the ability of a molecule to act or inhibit the protein.

## 3. Results

### 3.1. Construction of Compound and Target Protein Database

**Figure 1:**
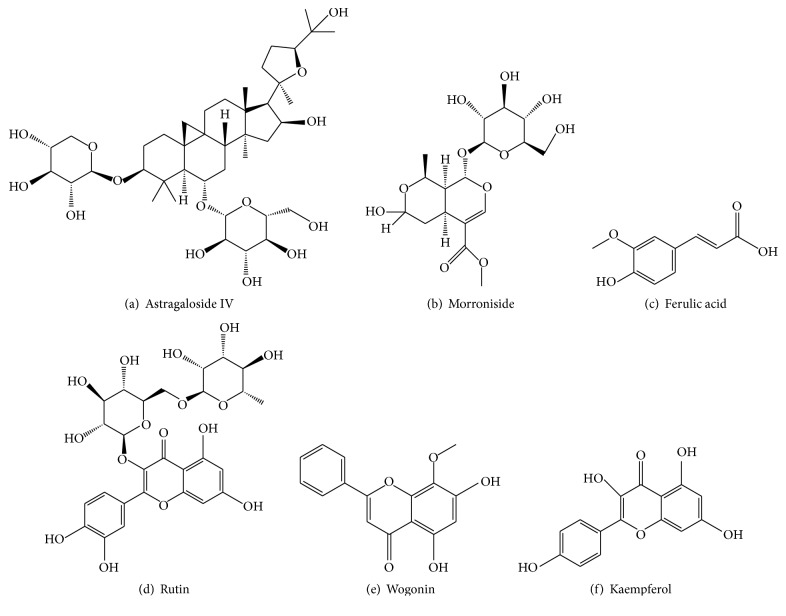
The structure of the six representative compounds.

From the constituents of GDC, we acquired 315 compounds for our compound database. Among these compounds, 72 were from* Astragali Radix*, 71 were from* Corni Fructus*, 33 were from* Radix Rehmanniae*, 23 were from* Sophorae Flos*, 60 were from* Scutellariae Radix*, 29 were from* Bombyx Batryticatus*, 13 were from* Leonuri Herba*, and 14 were from* Phyllanthi Fruit*. Finally, six representative compounds from this compound database were selected: astragaloside IV, morroniside, ferulic acid, rutin, wogonin, and kaempferol; their features are listed in Table 1 and the structures from PubMed are shown in Figure 1.

In the protein database, the proteins were classified by disease. There were 155 proteins in this database, which were divided into eight categories: diabetes mellitus (DM), special diabetes mellitus (special DM), diabetes mellitus type 1 (DM1), diabetes mellitus type 2 (DM2), nonrenal complications of diabetes mellitus (DM complication), diabetic nephropathy (DN), microvascular disease (MD), and diabetic microangiopathy. The principle of classification is based on the keyword of retrieval in Therapeutic Target Database. Finally, there were 49 proteins associated with DM, 13 proteins associated with DM1, 33 proteins associated with DM2, two proteins associated with special DM, four proteins associated with DM complication, 28 proteins associated with diabetic nephropathy, 13 proteins associated with MD, and 12 proteins associated with diabetic microangiopathy. The proteins are listed in Appendix 1 with their characteristic features. After the construction of the target protein database, more specific proteins parameters were sought. If there were pharmacological parameters of the RCSB Protein Data Bank available for the PDB code from UniProtKB, such as *Kd*, IC, and the action diagram with small molecules, they were used in subsequent procedures.

### 3.2. Target Prediction

**Figure 2:**
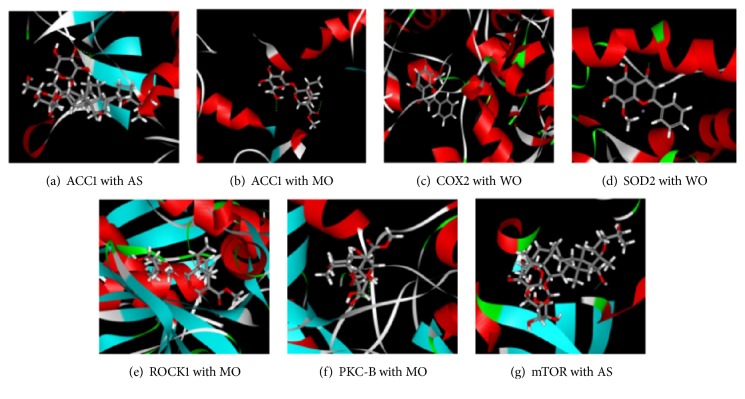
The docking results between the compounds and the proteins, respectively. Only the compound-protein pairs with valid results are shown.

In the docking study, the binding affinity between the molecule and protein was verified by comparing the LibDock score with the ligand. The molecules that had a higher LibDock score than the ligand were regarded to have a high affinity with protein; otherwise, they were considered to have a low affinity. In total, 99 proteins were examined for docking, and all results that could be exported to LibDock score are shown in Appendix. Finally, 47 target proteins had a higher LibDock score than the ligand, which meant that there was a high binding affinity between the molecules and proteins. The virtual docking of part of representative compound-protein pairs is shown in Figure 2.

From the analysis of the docking results, we identified that ferulic acid had the most target molecules, with 75 different valid results, followed by kaempferide with 69, morroniside and wogonin with 67, and rutin with 44. Astragaloside IV had the fewest targets, with only 34. However, morroniside was the most affiliative drug molecule of the six molecules with 37 high affinity results, whereas ferulic acid had only seven. For the other compounds, wogonin had 35 results, rutin had 25, kaempferide had 20, and astragaloside IV had 16.

Moreover, every molecule could dock with proteins with a high affinity for at least one protein in the different diseases, except astragaloside IV and rutin in DM1. Meanwhile, only RXRa could dock with high affinity to all six molecules. 25 proteins could dock with all six molecules with valid affinity, such as angiotensin-converting enzyme (ACE), integrin beta-7, and interstitial collagenase (MMP-1); 47 proteins could dock with at least one drug molecule with high affinity, such as acetyl-CoA carboxylase 1 (ACC1), glucagon receptor, protein kinase C, beta type (PKC-B), purinoceptor, tumor necrosis factor-alpha (TNF-*α*), transforming growth factor-*β*1 (TGF-*β*1), vasopressin receptor (V2R), aldosterone receptor (ALD), rho-associated protein kinase 1 (ROCK1), and superoxide dismutase [Mn] (SOD2).

### 3.3. Network Analysis

**Figure 3:**
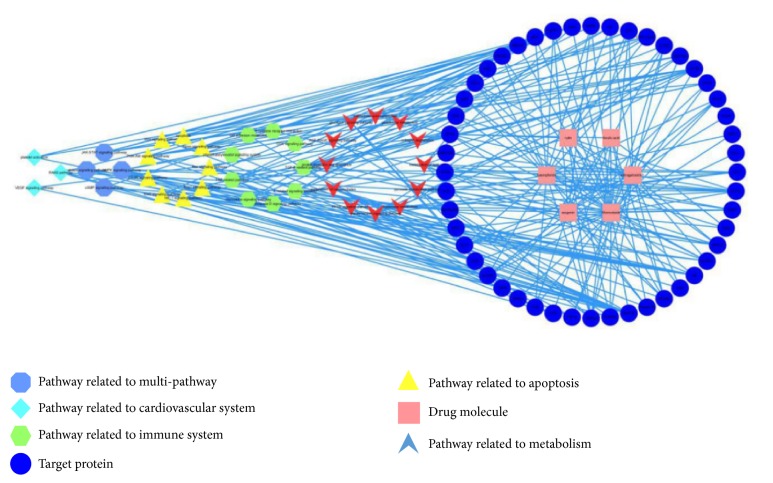
The compound-protein-pathway network diagram, including six compounds and 47 target proteins and their pathways.

**Figure 4:**
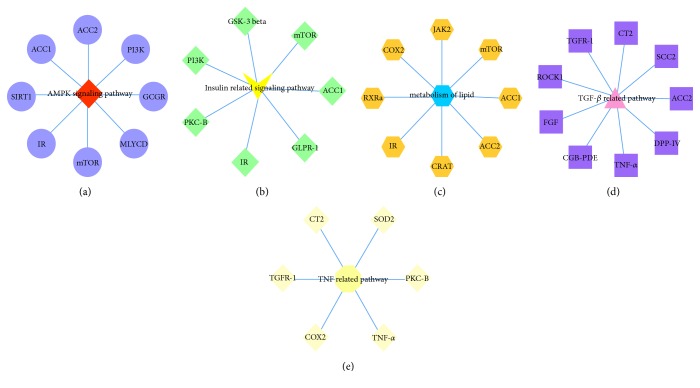
Five main pathways with their target proteins.

The networks based on the interaction between the protein, molecule, and pathway were shown in Figures 3 and 4.

There were 91 nodes and 250 edges in the protein-molecule-pathway network, and the proteins and drug molecules were independent terms. Meanwhile, according to the protein analysis, the interaction between these proteins and molecules that had high affinity could be classified into five different systems: immune system, cardiovascular system, apoptosis, metabolism, and multiple pathways. Different classifications were drawn in different colors and shapes. The centralization and heterogeneity of the network were 0.359 and 0.913, respectively.

There were 42 proteins related to metabolism, 33 related to the immune system, 32 related to apoptosis, 21 related to multiple pathways, and 10 related to cardiovascular system. Moreover, the TGF-related pathway (degree = 9) and the adenosine 5′-monophosphate- (AMP-) activated protein kinase (AMPK) signaling pathway (degree = 9) were linked with nine proteins, and the insulin-related signaling pathway (degree = 8) and lipid metabolism pathway (degree = 8) were linked with eight proteins and the TNF-related pathway (degree = 7) was linked with seven proteins; other pathways corresponded to at least one protein. Meanwhile, for the proteins, PKC-B was involved in the most pathways (15), IR was involved in 12, COX2 was involved in 10, and ROCK1, transforming growth factor receptor 1 (TGFR-1), and mTOR were involved in 9 each. Other proteins had a lower number of related pathways; for example, ACC1 was related to five pathways and SOD2 was related to four pathways.

### 3.4. Validity Assay of Target Protein

Seven protein-molecule pairs were selected to take part in the SPR test: ACC1 with astragaloside IV and morroniside, COX2 with astragaloside IV, morroniside and wogonin, and SOD2 with morroniside and wogonin. The results showed that astragaloside IV and morroniside had a high response and affinity with ACC1, with *Kd* values of 92.5 *μ*M and 13.63 *μ*M, respectively. In contrast, wogonin has a broader affinity with COX2 and SOD2, with *Kd* values of 44.67 *μ*M and 5.08 *μ*M, respectively. Three protein-molecule pairs, PKC-B with morroniside, ROCK1 with morroniside, and mTOR with astragaloside IV, were examined in the enzyme activity test. However, all compound-protein interactions were negative. The IC_50_ values of these protein-molecule pairs were more than 10000 *μ*M.

**Figure 5:**
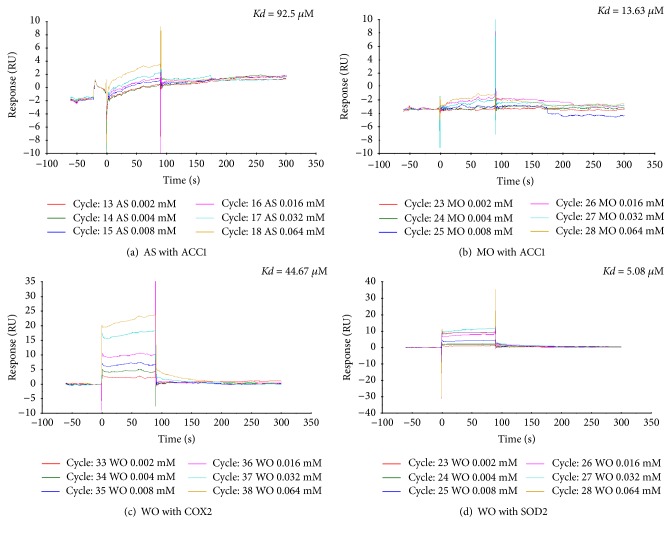
The results of SPR. Only compound-protein pairs with valid results are shown.

The information and results of proteins and their respective molecules are listed in Table 2. The results of SPR are shown in Figure 5.

## 4. Discussion

**Figure 6:**
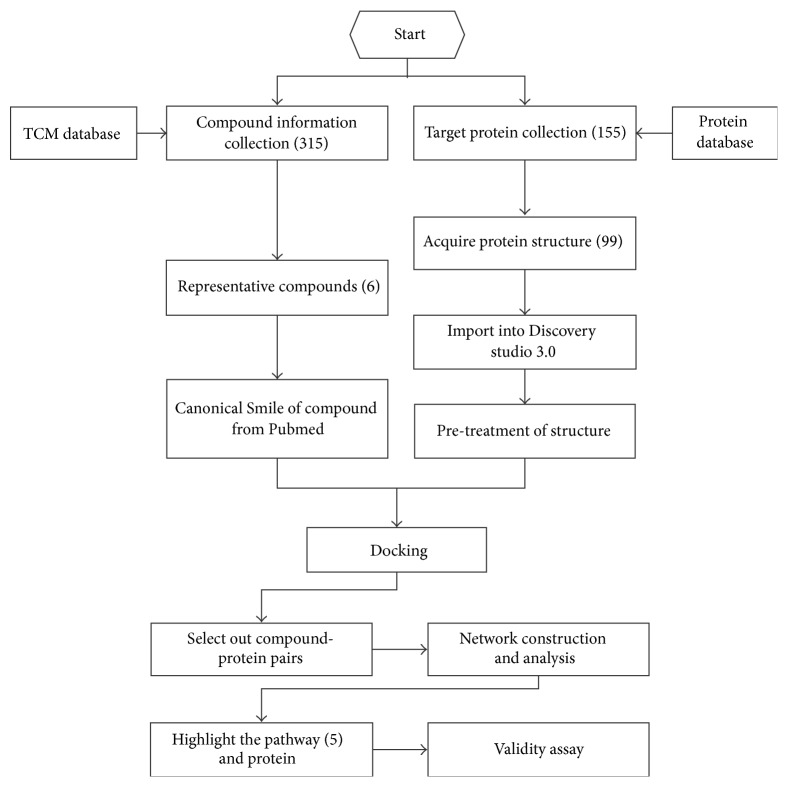
Flow chart of entire research.

TCM formulations are composed of multiple components with mechanisms of action that are always connected with multiple target proteins and pathways. GDC, a TCM formulation administered only in hospital, has been used to ameliorate microvascular complications in DM, such as diabetic nephropathy and diabetic retinopathy (DR), for some years. In the pharmaceutical analysis of GDC with HPLC-ESI-TOF/MS, 52 different components were identified, including morroniside, rutin, astragaloside IV, and wogonin. The biopharmaceutical analysis of drug molecules from GDC showed that 14 components, including morroniside, rutin, astragaloside IV, and wogonin, could be absorbed in blood after the oral administration of GDC, which means that they might act through blood circulation [12]. Given increased requirements for specific pharmacological mechanisms, we examined GDC in this study. The complete procedure of this research is presented in Figure 6.

The construction of the compound and target protein databases was the first step in our study. For the compound database, we collected compounds according to the herbs of GDC formula, obtained from Chemistry Database developed by Shanghai Organic Institute. To identify the main substances with pharmacological action in GDC, we used the following three rules for selection: (1) the rules of composition theory of the prescription in traditional Chinese medicine, (2) the quantity of substance in GDC, and (3) the hypoglycemic effect reported about diabetic nephropathy. Finally, the six representative compounds, astragaloside IV, morroniside, ferulic acid, rutin, wogonin, and kaempferol, were highlighted. To extend the database as much as possible, we chose the classification of diseases related to diabetic nephropathy as a criterion of acquiring target proteins; there were eight keywords for searching target proteins. The exact classifications were DM, DM1, DM2, special DM, DM complication, DN, MD, and diabetic microangiopathy. Finally, there are 155 proteins in our target protein database. Some studies have shown that many target proteins, such as angiotensin-converting enzyme (ACE), protein kinase C (PKC), and *α*-glucosidase, might be a therapeutic target for diabetic nephropathy [3]. These proteins were also identified in our target protein database. As can be seen, the protein database was shown to contain proteins covering different diseases of diabetic nephropathy, which was characteristic of extensive diversity that is supported by the complexity of TCM.

After the construction of the target protein database, 155 proteins were searched in UniProtKB; if they were identified in UniProtKB, the bias of the protein structure was compared with reality. After this procedure, 99 individual proteins were also found in the RCSB Protein Data Bank. The pharmacological parameters in RCSB Protein Data Bank, such as the *Kd*, IC, and action mechanism with small molecules, supported their reliable crystal structure for docking. For molecule docking, a high-throughput virtual screening approach was used. Then there were 47 proteins within our network analysis, and the degree of centrality was chosen to be the standard for influence of proteins or pathways in our study system. Finally, five representative pathways were defined, and 10 compound-protein pairs were examined in the validity assay. The methods above displayed a powerful capacity for the acquisition, processing, and integration of information on compounds in a TCM formulation; furthermore, it was specific to the complexity of the GDC capsule.

In this study, 99 proteins were evaluated for the molecular docking of six representative compounds. The results showed that morroniside was the most affiliative drug of the six molecules, with a high binding affinity with numerous proteins; furthermore, the action of morroniside may be clearer than that for other components. Ferulic acid could dock the most target molecules, but it also had the lowest number of high affinity results, indicating that the action of ferulic acid in the treatment of diabetic nephropathy occurs only because of the sum of various weak interactions. Other compounds also show affinities to different proteins, which may be the basis of their action as diabetic nephropathy therapeutics.

In this study, according to the result of molecular docking and network analysis, all protein-pathway pairs were distributed among metabolism, immune system, apoptosis, cardiovascular system, and multiple pathways. Moreover, the most frequent pathways were the TGF-related pathway and AMPK signaling pathway, which were linked with nine proteins, followed by the insulin-related signaling pathway, lipid metabolism, and the TNF-related pathway.

The TGF-related pathway mainly includes TGF pathway and TGFR pathway, in which TGF-*β* is a key mediator. TGF-*β* is active in human mesangial cells and causes oxidative stress and apoptosis in DN [15]. It also can regulate the Smad signaling pathway, which induces type I collagen synthesis in tubular epithelial cells and mesangial cells, ultimately leading to fibronectin in mesangial cells [16]. AMPK is a serine/threonine protein kinase that influences the process of DN through the regulation of energy metabolism, proliferation, inflammation, and apoptosis, in which the AMPK signaling pathway can also intervene [17]. Some proteins in the signaling pathway above, such as p38 MAPK and TGF-*β*, can regulate the AGE-RAGE pathway, which activates multiple signaling pathways implicated in inflammation, cancer, and other diseases [18]. That is, the AMPK signaling pathway and the TGF-related pathway may have potential for synergistic action in the development of DN.

The insulin-related signaling pathway, which has an array of mediators, including IR and GLPR-1, is active in the process of DN. The pathway of lipid metabolism covers eight proteins, such as ACC1, ACC2, IR, and mTOR, which can regulate the generation, decomposition, or actions of lipid. A study indicated that dyslipidemia was a risk factor in the process of kidney disease in type 2 diabetes mellitus and that the accumulation of lipid causes podocyte dysfunction and apoptosis in DN [19].

TNF-*α* is one of the most active proteins in the TNF-related pathway, which may be activated in inflammation development. The research by Guo et al. showed that the presence of infiltrating macrophages in DN promotes podocyte apoptosis via TNF-*α*-related pathway [20]. Presently, DN has been defined as the occurrence of a series of abnormal changes in pathophysiology, including metabolic disorder, oxidative stress, inflammatory processes, apoptosis, and hemodynamic changes [3, 21, 22]. In addition, based on the information from KEGG, the main pathways of DN are AGE-RAGE signaling pathway, renin-angiotensin system (RAS), FoxO signaling pathway, and longevity regulation. Some common pathways also exist in this pathway, such as the AMPK pathway, TNF-*α* signaling pathway, and TGF signaling pathway.

In addition to these five main pathways above, some pathways related to apoptosis were also found in our investigation, such as the Ras signaling pathway, the Rap1 signaling pathway, and the Wnt signaling pathway. It appears that many pairs of protein and molecules can influence these pathways, such as PKC-B with morroniside, FGF with astragaloside IV and morroniside, and GSK-3*β* with rutin. Moreover, protein metabolism may be another approach for the treatment of GDC. However, only a small number of proteins are involved in this way, such as DPP-IV and NEP. Meanwhile, as for RAS, some proteins are involved with compounds, such as V2R, ALD, and NEP. ALD has been defined as a regulatory activator of sodium and water, which is unbalanced in DN [3].

As for proteins, the centrality degree of proteins in the network analysis and their evidence of influence for the process of DN have been considered. Ultimately, PKC-B, COX2, ROCK1, mTOR, SOD2, and ACC1 were chosen to be the representative compounds. In combination with the information extracted from the five pathways above, we found that ACC1 might influence the AMPK signaling pathway, the insulin-related signaling pathway, and lipid metabolism; mTOR could influence the AMPK signaling pathway, the insulin-related signaling pathway, and lipid metabolism; COX2 could alter lipid metabolism and the TNF-related pathway; PKC-B could participate in the insulin-related signaling pathway and the TNF-related pathway. SOD2 influenced the TGF-related and TNF-related pathways and ROCK1 influenced the TGF-related pathway. According to the pathway information, the action of ACC1 and mTOR focused on the metabolism of glucose and lipids, inflammation, and apoptosis in DN, whereas PKC-B and COX2 were concentrated on metabolism and inflammation. SOD2 altered oxidative stress and apoptosis and ROCK1 mainly influenced apoptosis. Collectively, the actions of these proteins are concentrated on metabolism, inflammation, apoptosis, and oxidative stress in DN.

Moreover, the results of the validity assay indicated that the action of ACC1 can be influenced by astragaloside IV and morroniside, whereas COX2 and SOD2 can be altered by wogonin.

Astragaloside IV can regulate the AMPK signaling pathway, the insulin-related signaling pathway, and the metabolism of lipids through an influence on the activity of ACC1. The research of Zhang showed that astragaloside IV altered the NF-kappa B pathway to counteract the expression of E-selectin and VCAM-1 in response to inflammatory mediators, which meant that it has credible anti-inflammatory ability [23]. Combined with our study, the anti-inflammatory action of astragaloside IV may occur through the changes in ACC1.

While morroniside can take part in the TGF-related pathway, the insulin-related signaling pathway and the TNF-related pathway may be altered by ROCK1 and PKC-B. An early study pointed out that morroniside alleviated oxidative stress under diabetic pathological conditions through a reduction in ROS level and the downregulation of the expression of iNOS and COX2, followed by the inhibition of NF-*κ*B transcription [24]. Some research has shown that morroniside can alter the AGE-RAGE pathway to suppress the high expression of p38 MAPK and the synthesis and release of NF-kB, ICAM-1, MCP-1, and TGF-*β* [18]. Thus, morroniside may have an effect through the alteration of ROCK1 and PKC-B in the TGF-related pathway, insulin-related signaling pathway, and the TNF-related pathway.

Wogonin can affect the TGF-related pathway, lipid metabolism, and TNF-related pathway through the regulation of COX2 and SOD2. Clearly, anti-inflammation may be a method of action of wogonin, and a study demonstrated that wogonin inhibited high-glucose-induced vascular inflammation, which was attributed to antioxidant ability and the inhibition of LPS-induced nitric oxide (NO) production and the expression of the nitric oxide synthase (iNOS) gene [25]. According to our research, wogonin affected the progress of inflammation in DN through the regulating of COX2 and SOD2, so that it can regulate the TGF-related pathway, lipid metabolism, and TNF-related pathway.

In conclusion, our research demonstrated that these six representative compounds in GDC exert a potential influence on the pathways that are disordered in diabetic nephropathy through their constituent proteins. However, the results are only based on virtual methods; based on the results of SPR and the enzyme activity test, further research is required to reveal the actual action of GDC in diabetic nephropathy.

## 5. Conclusions

In this study, six representative compounds in GDC were chosen to dock with 155 proteins related to diabetic nephropathy. As a result, 47 proteins were identified with a high affinity with at least one compound molecule. These 47 proteins were analyzed in the protein-molecule-pathway network. It was found that the TGF-*β*1 pathway, AMPK signaling pathway, insulin-related signaling pathway, lipid metabolism, and TNF-*α* pathway were the major pathways of treatment of GDC for diabetic nephropathy. These five pathways may be the primary aspects of the treatment for GDC in diabetic nephropathy. Moreover, astragaloside IV and morroniside were shown to alter ACC1 directly (*Kd* = 92.5 *μ*M and 13.63 *μ*M), whereas wogonin could bind with COX2 and SOD2 (*Kd* = 44.67 *μ*M and 5.08 *μ*M).

In summary, the action of GDC in the treatment of diabetic nephropathy was concentrated on metabolism regulation, anti-inflammation, the inhibition of apoptosis, and exerted pharmaceutical actions based on the regulation of the medial proteins in these pathways.

## Figures and Tables

**Table 1:** The information of drug molecules.

ID	Compounds	Short name	Molecular formula	Molecular weight (g·mol^−1^)	Origin	Classification
1	Astragaloside IV	AS	C_41_H_68_O_14_	784.9702	*Astragali Radix*	Glucoside
2	Morroniside	MO	C_17_H_26_O_11_	406.3840	*Corni Fructus*	Glucoside
3	Ferulic acid	FA	C_10_H_10_O_4_	194.1840	*Corni Fructus*	Phenolic acid
4	Rutin	RU	C_27_H_30_O_16_	610.5175	*Sophorae Flos/Leonuri Herba*	Flavonoid
5	Wogonin	WO	C_22_H_20_O_11_	460.3876	*Scutellariae Radix*	Glucosides
6	Kaempferide	KA	C_16_H_12_O_6_	300.2629	*Astragali Radix/Phyllanthi Fruit*	Sugar acids

**Table 2:** Information and results of the target protein in the validity assay.

Number	Full name of protein	Short name of protein	Respective molecule	Test method	Results of experiment			
					*Kd* (*μ*M)	*R*max (RU)	Offset (RU)	*χ* ^2^ (RU^2^)
1	Acetyl-CoA carboxylase 1	ACC1	Astragaloside IV	SPR	92.5	7.856	0.2424	0.0243
			Morroniside	SPR	13.63	2.204	−0.2651	0.0962

2	Cyclooxygenase-2	COX2	Astragaloside IV	SPR				
			Morroniside	SPR	44.67	39.54	0.6314	0.799
			Wogonin	SPR				

3	Superoxide dismutase [Mn]	SOD2	Morroniside	SPR				
			Wogonin	SPR	5.08	14.3	−3.611	2.11

					IC50 (*μ*M)			

4	Protein kinase C, beta type	PKC-B	Morroniside	EMSA	>10000			

5	Rho-associated protein kinase 1	ROCK1	Morroniside	EMSA	>10000			

6	Mammalian target of rapamycin	mTOR	Astragaloside IV	Lance ultra	>10000			

## Data Availability

The data of our research can be acquired from the Supplementary Materials uploaded with this article.

## Supplementary Materials

Supplementary Materials Six files have been uploaded as supplementary materials for this research. The first file is the WORD file of the appendix, which has two forms. One is the target protein database, and the other is the form of valid results between the compounds and proteins in docking. The second file is an EXCEL file of the compound database including 315 compounds. The third file is the graphical abstract in PDF, which shows the main process of the research with pictures. The fourth file is a form with three sheets in EXCEL format, which includes total results of docking, valid results of docking, and high affinity results of docking. Meanwhile, the fifth file and the sixth file are EXCEL files of the network diagram of representative target-compound networks and target-compound-pathway networks, respectively.
