# Supplementary material for: Revealing Synergistic Mechanism of Multiple Components in Gandi Capsule for Diabetic Nephropathy Therapeutics by Network Pharmacology
Source: Evid Based Complement Alternat Med. 2018 Apr 26;2018:6503126. doi: 10.1155/2018/6503126 (PMC5944259; doi:10.1155/2018/6503126)
Supplement: Supplementary Materials — Six files have been uploaded as supplementary materials for this research. The first file is the WORD file of the appendix, which has two forms. One is the target protein database, and the other is the form of valid results between the compounds and proteins in docking. The second file is an EXCEL file of the compound database including 315 compounds. The third file is the graphical abstract in PDF, which shows the main process of the research with pictures. The fourth file is a form with three sheets in EXCEL format, which includes total results of docking, valid results of docking, and high affinity results of docking. Meanwhile, the fifth file and the sixth file are EXCEL files of the network diagram of representative target-compound networks and target-compound-pathway networks, respectively. [file 6503126.f1.zip › 6503126.f1/appendix.docx]

| NO. | target name | disease | PDB code |
| --- | --- | --- | --- |
| 1 | Acetyl-CoA carboxylase1 | DM | 3COJ |
| 2 | Acetyl-CoA carboxylase2 | DM | 3JRX |
| 3 | AMP-activated protein kinase | DM | 4RER |
| 4 | ATP-sensitive inward rectifier potassium channel 10 | DM |  |
| 5 | ATP-sensitive K+ channel | DM |  |
| 6 | Beta-secretase 2 | DM | 2EWY |
| 7 | Carnitine acyltransferase | DM | 1s5o |
| 8 | Dipeptidyl peptidase IV | DM | 2G63 |
| 9 | Forkhead box O | DM |  |
| 10 | Free fatty acid receptor1 | DM | 4PHU |
| 11 | Fructosamine oxidase | DM | 3DJE |
| 12 | Ghrelin receptor | DM |  |
| 13 | Glucagon-like peptide 1 receptor | DM | 3C59 |
| 14 | Glucocorticoid receptor | DM | 4P6W |
| 15 | Glucokinase | DM | 4DHY |
| 16 | Cytosolic β-Glucosidase | DM | 2E9L |
| 17 | Glycogen synthase | DM |  |
| 18 | GPR119 | DM |  |
| 19 | G-protein coupled receptor-39 | DM |  |
| 20 | Insulin-like Growth Factor1 | DM | 1imx |
| 21 | Insulin-like Growth Factor2 | DM | 2v5p |
| 22 | insulin receptor | DM | 4IBM |
| 23 | Interleukin-2 | DM | 1M48 |
| 24 | Ketohexokinase | DM | 3QAI |
| 25 | Leptin receptor | DM | 3v6o |
| 26 | Hepatic triacylglycerol lipase | DM |  |
| 27 | Maltase-glucoamylase | DM | 2QMJ |
| 28 | Munc13 | DM |  |
| 29 | Pancreatic beta cell growth factor | DM |  |
| 30 | Pepsin 3A | DM | 1F34 |
| 31 | Peptide YY ligand | DM |  |
| 32 | Peroxisome proliferator activated receptor delta | DM | 3GZ9 |
| 33 | Peroxisome proliferator activated receptor gamma | DM | 4R6S |
| 34 | Peroxisome proliferator activated receptor | DM |  |
| 35 | Phosphodiesterase Type 10 | DM | 3UI7 |
| 36 | Platelet-derived growth factor receptorα | DM | 5K5X |
| 37 | Platelet-derived growth factor receptorβ | DM | 1SHA |
| 38 | Prostaglandin receptor | DM |  |
| 39 | Protein Kinase C, beta Type | DM | 2I0E |
| 40 | Protein tyrosine phosphatase-1B | DM | 1LQF |
| 41 | Pyruvate dehydrogenase kinase isoform 2 | DM | 2BU5 |
| 42 | Recombinant human angiotensin converting enzyme 2 | DM | 1R4L |
| 43 | RXRa | DM | 1FM6 |
| 44 | Serine/threonine protein kinase | DM |  |
| 45 | SUR1-type K(ATP) channel | DM |  |
| 46 | SUR2-type K(ATP) channel | DM |  |
| 47 | Tumor necrosis factor alpha | DM | 4TWT |
| 48 | Vasopressin receptor | DM | 4JQI |
| 49 | Zinc alpha-2 glycoprotein precursor | DM |  |
| 50 | 5-hydroxytryptamine 2A receptor | DM |  |
| DM1 |  |  |  |
| NO. | target name | disease | PDB code |
| 1 | CD6 | DM1 | 5A2E |
| 2 | Ghrelin | DM1 |  |
| 3 | Glutamate decarboxylase 2 | DM1 | 2OKK |
| 4 | HLA class II antigen DQ-2 alpha | DM1 | 1UVQ |
| 5 | Islet-specific glucose-6-phosphatase catalytic subunit-related protein | DM1 |  |
| 6 | Lysophosphatidic acid transferase1 | DM1 |  |
| 7 | Lysophosphatidic acid transferase2 | DM1 |  |
| 8 | Natural cytotoxic triggering receptor 1 | DM1 |  |
| 9 | Neuropeptide recptor | DM1 |  |
| 10 | Proprotein convertase subtilisin/kexin type 9 | DM1 | 2QTW |
| 11 | Protein tyrosine phosphatase | DM1 | 5BZX |
| 12 | Regulatory protein | DM1 |  |
| 13 | T-cell surface glycoprotein CD3 epsilon chain | DM1 | 1A81 |
| DM2 |  |  |  |
| NO. | target name | disease | PDB code |
| 1 | Bile-salt-activated lipase | DM2 | 1F6W |
| 2 | Connective tissue growth factor | DM2 |  |
| 3 | Corticosteroid 11-beta-dehydrogenase, isozyme 1 | DM2 | 3BZU |
| 4 | Corticosteroid 11-beta-dehydrogenase, isozyme 2 | DM2 |  |
| 5 | D1 dopamine receptor-interacting protein calcyon | DM2 |  |
| 6 | Estrogen related receptor alpha | DM2 | 2PJL |
| 7 | Fetuin | DM2 |  |
| 8 | FGF-21 ligand | DM2 |  |
| 9 | Fructose-1,6-bisphosphatase1 | DM2 | 1FTA |
| 10 | Fructose-1,6-bisphosphatase2 | DM2 | 3IFC |
| 11 | Gastric inhibitory polypeptide receptor | DM2 | 2QKH |
| 12 | Gastrin-releasing peptide receptor | DM2 |  |
| 13 | Glucagon receptor | DM2 | 5ee7 |
| 14 | Glucagon-like peptide 1 | DM2 | 4ZGM |
| 15 | Glucose-dependent insulinotropic receptor | DM2 |  |
| 16 | Glycogen phosphorylase, muscle form | DM2 | 1z8d |
| 17 | Free fatty acid receptor 2 | DM2 |  |
| 18 | Interferon beta ligand | DM2 | 1au1 |
| 19 | Interleukin-18 | DM2 | 3WO4 |
| 20 | Interleukin-6 receptor | DM2 | 1N26 |
| 21 | Lysosomal alpha-glucosidase | DM2 |  |
| 22 | Melanocortin-3 receptor | DM2 |  |
| 23 | MTP | DM2 |  |
| 24 | PI3K gamma | DM2 | 3L54 |
| 25 | Purinoceptor | DM2 | 4XNV |
| 26 | SGLT2 | DM2 |  |
| 27 | Sirtuin1 | DM2 | 4I5I |
| 28 | Sirtuin2 | DM2 | 5d7p |
| 29 | Sodium dependent glucose transport | DM2 |  |
| 30 | Sodium/glucose cotransporter 2 | DM2 |  |
| 31 | Solute carrier family 2, facilitated glucose transporter, member 4 | DM2 |  |
| 32 | Stearoyl CoA desaturase-1 | DM2 | 4YMK |
| 33 | TGR5 | DM2 |  |
| special DM |  |  |  |
| NO. | target name | disease | PDB code |
| 1 | Insulin-like growth factor binding protein 1 | special DM | 2dsq |
| 2 | Toll-like receptor 2 | special DM | 2Z7X |
| DM complication |  |  |  |
| NO. | target name | disease | PDB code |
| 1 | Alcohol dehydrogenase | Diabetic complication | 2ALR |
| 2 | Caveolin-1 | Diabetic complication |  |
| 3 | Cyclin-dependent kinase inhibitor 1C | Diabetic complication |  |
| 4 | Vascular adhesion protein 1 | Diabetic complication | 1VCA |
| DN |  |  |  |
| NO. | target name | disease | PDB code |
| 1 | Peroxiredoxin 4 | DN | 3TKS |
| 2 | Adenosine A2a receptor | DN | 3EML |
| 3 | Aldose reductase | DN | 1EF3 |
| 4 | Aldosterone receptor | DN | 2AA2 |
| 5 | Angiotensin-converting enzyme | DN | 2OC2 |
| 6 | C-C chemokine receptor type 5 | DN | 4MBS |
| 7 | CGMP-specific 3',5'-cyclic phosphodiesterase | DN | 1tbf |
| 8 | Cyclooxygenase1 | DN |  |
| 9 | Cyclooxygenase2 | DN | 5F1A |
| 10 | Endothelin-1 receptor | DN |  |
| 11 | Fibroblast growth factor 1 | DN | 4qo3 |
| 12 | Heat shock protein 70 1A | DN | 3atu |
| 13 | Heat shock protein 70 1B | DN | 4j8f |
| 14 | heparanase | DN | 5e9c |
| 15 | Interleukin-22 | DN | 3dlq |
| 16 | Interstitial collagenase | DN | 1cgl |
| 17 | JAK1 | DN | 3eyg |
| 18 | Kallikrein | DN | 1spj |
| 19 | MAP kinase p38 | DN | 1a9u |
| 20 | GLIPR-2 | DN | 4aiw |
| 21 | Mitogen-activated protein kinase kinase kinase 5 | DN | 2clq |
| 22 | NADPH oxidase-NOx5 | DN |  |
| 23 | Neprilysin | DN | 1dmt |
| 24 | Rho-associated protein kinase 1 | DN | 2etk |
| 25 | soluble epoxide hydrolase | DN | 1vj5 |
| 26 | TGF-beta receptor type I | DN | 1py5 |
| 27 | Tyrosine-protein kinase JAK2 | DN | 2b7a |
| 28 | Protein Kinase C,αType | DN | 3IW4 |
| MD |  |  |  |
| NO. | target name | disease | PDB code |
| 1 | A disintegrin and metalloproteinase with thrombospondin motifs 4 | MD | 2rjp |
| 2 | Aminopeptidase N | MD | 4fyr |
| 3 | Amyloid beta A4 protein | MD | 5csz |
| 4 | Cathepsin D | MD | 4obz |
| 5 | Copper-transporting ATPase 2 | MD | 3cjk |
| 6 | Copper-transporting ATPase1 | MD |  |
| 7 | Glycogen synthase kinase-3 beta | MD | 1q3d |
| 8 | G-protein coupled estrogen receptor 1 | MD |  |
| 9 | Hypoxia-inducible factor 1-alpha | MD | 3hqu |
| 10 | Integrin beta-7 | MD | 3v4v |
| 11 | Matrix metalloproteinase-19 | MD |  |
| 12 | Protein DJ-1 | MD | 3cza |
| 13 | Puromycin-sensitive aminopeptidase | MD |  |
| DN,MD |  |  |  |
| NO. | target name | disease | PDB code |
| 1 | 11-beta-hydroxysteroid dehydrogenase， isozyme 2 | DN,MD |  |
| 2 | Diacylglycerol O-acyltransferase 1 | DN,MD |  |
| 3 | Interleukin-1 receptor antagonist protein | DN,MD | 1IRA |
| 4 | Malonyl-CoA decarboxylase, mitochondrial | DN,MD | 4f0x |
| 5 | Mammalian target of Rapamycin | DN,MD | 1fap |
| 6 | NADPH oxidase | DN,MD |  |
| 7 | Serum paraoxonase/arylesterase 1 | DN,MD | 1v04 |
| 8 | Solute carrier family 22 member 6 | DN,MD |  |
| 9 | Stearoyl-CoA desaturase 5 | DN,MD |  |
| 10 | Superoxide dismutase [Mn] | DN,MD | 2adp |
| 11 | Vascular endothelial growth factor A | DN,MD | 3QTK |
| 12 | Voltage-gated potassium channel | DN,MD |  |

Appendix 1:the information of proteins

| NO. | target name | short name of target | PDB code | disease | astragalosideⅣ | morroniside | ferulic acid | rutin | wogonin | kaempferide | ligand |
| --- | --- | --- | --- | --- | --- | --- | --- | --- | --- | --- | --- |
| 1 | Acetyl-CoA carboxylase 1 | ACC1 | 3COJ | DM | 100.229↑ | 126.543↑ | 46.7383↓ |  | 93.8182↑ | 107.165↑ | 90.5333 |
| 2 | Acetyl-CoA carboxylase 2 | ACC2 | 3JRX | DM | 162.36↑ | 135.974↑ | 66.6798↓ | 137.501↑ | 97.8305↓ | 104.002↓ | 113.5880 |
| 3 | AMP-activated protein kinase | AMPK | 4RER | DM |  | 118.702↓ | 55.0136↓ | 106.526↓ | 91.6604↓ | 96.4313↓ | 142.7620 |
| 4 | Beta-secretase 2 | BACE2 | 2EWY | DM |  | 135.629↓ | 58.7846↓ |  | 93.6622↓ | 97.4896↓ | 165.2120 |
| 5 | Carnitine acyltransferase | CRAT | 1s5o | DM |  | 118.122↑ | 58.0263↓ |  | 89.042↑ | 86.1139↑ | 62.9424 |
| 6 | Cytosolic β-Glucosidase | CBG | 2E9L | DM |  |  | 68.419↑ |  |  | 91.0048↑ | 40.7202 |
| 7 | Dipeptidyl peptidase IV | DPP-IV | 2G63 | DM | 151.06↑ | 125.422↑ | 55.5065↓ | 160.657↑ | 85.6902↓ | 106.226↓ | 123.0000 |
| 8 | Free fatty acid receptor1 | FFAR1 | 4PHU | DM | 65.9737↓ | 98.3158↓ | 73.9336↓ |  | 96.2661↓ | 116.783↓ | 161.6300 |
| 9 | Fructosamine oxidase | FAOX | 3DJE | DM |  |  | 57.9381↓ |  | 114.108↑ | 85.0907↓ | 100.2140 |
| 10 | Glucagon-like peptide 1 receptor | GLPR-1 | 3C59 | DM | 117.317↑ | 113.393↑ | 60.2452↓ |  | 76.9067↑ | 95.7965↑ | 75.4985 |
| 11 | Glucocorticoid receptor | GR | 4P6W | DM |  | 119.783↓ | 58.6039↓ |  | 84.8228↓ | 97.1514↓ | 145.5950 |
| 12 | Glucokinase | GK | 4DHY | DM |  |  | 75.964↓ |  |  |  | 101.5480 |
| 13 | Insulin-like Growth Factor2 | IGF2 | 2v5p | DM |  | 92.4891↓ | 46.1357↓ |  | 64.7078↓ |  | 97.4003 |
| 14 | insulin receptor | IR | 4IBM | DM |  | 121.946↑ | 52.1252↓ |  | 87.4264↓ | 92.1442↓ | 117.2660 |
| 15 | Interleukin-2 | IL-2 | 1M48 | DM | 126.64↓ | 103.383↓ | 62.7339↓ | 96.0068↓ | 78.892↓ | 89.8972↓ | 139.0540 |
| 16 | Ketohexokinase | KHK | 3QAI | DM | 147.267↑ | 132.091↓ | 66.8968↓ | 149.847↑ | 97.1415↓ | 102.142↓ | 138.5890 |
| 17 | Maltase-glucoamylase | MGAM | 2QMJ | DM | 124.069↑ | 110.239↑ | 62.4124↑ |  | 67.3473↑ | 78.4625↑ | 53.0502 |
| 18 | Pepsin 3A | Pepsin 3A | 1F34 | DM |  |  | 65.7352↑ |  | 92.3467↑ | 106.415↑ | 53.9336 |
| 19 | Peroxisome proliferator activated receptor delta | PPAR-delta | 3GZ9 | DM | 92.719↓ | 101.016↓ | 48.5297↓ | 87.9119↓ | 73.5728↓ | 82.7219↓ | 108.0460 |
| 20 | Phosphodiesterase Type 10 | PDE10 | 3UI7 | DM |  |  |  |  |  | 102.011↓ | 109.3710 |
| 21 | Protein Kinase C, beta Type | PKC-B | 2I0E | DM |  | 113.553↑ | 51.3655↓ | 91.2446↓ | 88.0759↓ | 97.4258↓ | 109.5300 |
| 22 | Protein tyrosine phosphatase-1B | PTP-1B | 1LQF | DM | 127.21↓ | 117.973↓ | 72.3907↓ |  | 86.8955↓ | 85.1252↓ | 201.0260 |
| 23 | Pyruvate dehydrogenase kinase isoform 2 | PDHK2 | 2BU5 | DM | 154.463↑ | 124.082↑ | 71.6419↓ | 124.764↑ | 93.987↓ | 102.147↓ | 113.1910 |
| 24 | Recombinant human angiotensin converting enzyme 2 | ACE2 | 1R4L | DM | 113.761↓ | 101.648↓ | 50.5277↓ | 100.411↓ | 69.7827↓ | 74.6327↓ | 114.1980 |
| 25 | RXRa | RXRa | 1FM6 | DM | 130.447↑ | 105.831↑ | 52.8218↑ | 75.7732↑ | 77.5379↑ | 94.5362↑ | -24.9156 |
| 26 | Tumor necrosis factor alpha | TNF-α | 4TWT | DM | 140.38↑ | 130.746↑ | 53.4364↓ | 96.7117↓ | 89.0808↓ | 101.81↓ | 129.8200 |
| 27 | Vasopressin receptor | V2R | 4JQI | DM |  |  | 51.1809↑ |  |  |  | 41.5811 |
| 28 | CD6 | CD6 | 5A2E | DM1 |  | 102.238↑ | 50.2612↓ |  | 66.4694↑ | 82.5974↑ | 50.6248 |
| 29 | Glutamate decarboxylase 2 | GAD2 | 2OKK | DM1 |  |  | 33.5595↓ |  |  |  | 48.6495 |
| 30 | HLA class II antigen DQ-2 alpha | DC-α | 1UVQ | DM1 |  | 61.2856↓ | 41.1495↓ |  |  |  | 66.6006 |
| 31 | Protein tyrosine phosphatase | PTP | 5BZX | DM1 |  |  | 88.2226↓ |  |  |  | 93.5106 |
| 32 | Corticosteroid 11-beta-dehydrogenase, isozyme 1 | 11-DH | 3BZU | DM2 |  | 144.217↓ | 73.5715↓ | 111.1↓ | 101.101↓ | 124.59↓ | 210.9080 |
| 33 | Estrogen related receptor alpha | ERR-α | 2PJL | DM2 |  | 123.235↑ | 58.7158↓ |  | 91.9818↓ | 92.6012↓ | 121.6470 |
| 34 | Fructose-1,6-bisphosphatase1 | FBP1 | 1FTA | DM2 |  | 121.962↓ | 67.7439↓ | 114.141↓ | 103.958↓ | 118.547↓ | 126.7230 |
| 35 | Fructose-1,6-bisphosphatase2 | FBP2 | 3IFC | DM2 |  | 93.412↓ | 62.5216↓ |  | 74.6934↓ | 74.2649↓ | 98.6807 |
| 36 | Gastric inhibitory polypeptide receptor | GIPR | 2QKH | DM2 |  |  | 41.043↑ |  |  |  | 31.8769 |
| 37 | Glucagon receptor | GCGR | 5ee7 | DM2 | 101.234↑ | 129.837↑ | 69.5017↓ | 72.3559↓ | 108.795↑ | 106.871↑ | 77.4428 |
| 38 | Interleukin-6 receptor | IL-6R | 1N26 | DM2 | 100.213↓ | 75.7484↓ |  | 108.825↓ |  | 52.2145↓ | 111.3660 |
| 39 | PI3K gamma | PI3K | 3L54 | DM2 |  | 119.986↑ | 65.7294↓ |  | 96.7761↓ | 102.082↓ | 106.2900 |
| 40 | Purinoceptor | P2Y | 4XNV | DM2 | 82.7257↑ | 82.9886↑ | 46.3282↓ | 60.0648↓ | 79.3589↑ | 85.2713↑ | 68.9713 |
| 41 | Sirtuin1 | SIRT1 | 4I5I | DM2 |  | 151.069↑ | 68.0519↓ |  | 104.604↓ | 105.925↓ | 106.7410 |
| 42 | Sirtuin2 | SIRT2 | 5d7p | DM2 |  | 146.788↑ | 84.7121↓ |  | 103.49↓ | 125.865↑ | 114.9380 |
| 43 | Stearoyl CoA desaturase-1 | SCD-1 | 4YMK | DM2 |  |  | 59.2918↓ |  |  | 133.024↓ | 163.0150 |
| 44 | Adenosine A2a receptor | ADORA2A | 3EML | DN |  | 126.168↑ | 57.4898↓ |  | 93.9516↓ | 101.531↓ | 106.4000 |
| 45 | Aldose reductase | AR | 1EF3 | DN |  |  | 79.144↓ |  | 114.61↓ | 105.744↓ | 143.3790 |
| 46 | Aldosterone receptor | ALD | 2AA2 | DN | 76.9998↓ | 85.216↑ | 36.7392↓ | 61.4232↓ | 57.0466↓ | 85.2808↑ | 84.4052 |
| 47 | Angiotensin-converting enzyme | ACE | 2OC2 | DN | 140.184↓ | 109.701↓ | 45.6454↓ | 98.6061↓ | 76.655↓ | 97.1026↓ | 142.3120 |
| 48 | C-C chemokine receptor type 5 | CCR5 | 4MBS | DN | 154.803↑ | 124.615↑ | 63.5443↓ | 120.506↑ | 88.0354↑ |  | 81.9938 |
| 49 | CGMP-specific 3',5'-cyclic phosphodiesterase | CGB-PDE | 1tbf | DN | 154.391↑ | 132.357↓ | 61.4627↓ | 123.808↓ | 96.187↓ | 111.428↓ | 139.8310 |
| 50 | Cyclooxygenase2 | COX2 | 5F1A | DN | 160.525↑ | 140.735↑ | 75.3506↑ | 141.242↑ | 110.774↑ |  | 38.0689 |
| 51 | Fibroblast growth factor 1 | FGF | 4qo3 | DN | 99.4409↑ | 115.608↑ | 45.0967↓ | 61.1299↓ | 54.9364↓ | 56.2241↓ | 65.8995 |
| 52 | Heat shock protein 70 1A | HSF1A | 3atu | DN |  | 150.855↓ | 73.9036↓ |  | 115.027↓ | 120.906↓ | 163.4960 |
| 53 | Heat shock protein 70 1B | HSF1B | 4j8f | DN |  | 148.422↓ | 69.7487↓ |  | 124.003↓ | 124.46↓ | 159.9410 |
| 54 | heparanase | HEP | 5e9c | DN | 143.566↓ | 126.912↓ | 64.5137↓ | 122.545↓ | 82.2224↓ | 91.414↓ | 191.4810 |
| 55 | Interstitial collagenase | MMP-1 | 1cgl | DN | 161.873↓ | 141.154↓ | 77.8062↓ | 128.195↓ | 92.9515↓ | 107.386↓ | 205.2270 |
| 56 | JAK1 | JAK1 | 3eyg | DN |  | 126.035↑ | 55.9754↓ |  | 99.8534↓ | 108.362↑ | 103.5810 |
| 57 | Kallikrein | KLK1 | 1spj | DN | 123.027↑ | 108.786↑ | 61.6694↓ | 86.5483↓ | 88.2672↓ | 110.188↑ | 95.1073 |
| 58 | MAP kinase p38 | MAPK12 | 1a9u | DN |  | 110.818↓ | 54.907↓ | 100.354↓ | 77.1262↓ | 85.3087↓ | 131.5830 |
| 59 | Mitogen-activated protein kinase kinase kinase 5 | MAP3K5 | 2clq | DN |  | 108.767↓ | 48.5121↓ | 128.081↓ | 88.732↓ | 91.1073↓ | 136.7290 |
| 60 | Neprilysin | NEP | 1dmt | DN |  |  |  |  |  | 109.557↑ | 61.4516 |
| 61 | Protein Kinase C,αType | PKC-A | 3IW4 | DN |  | 122.598↓ | 54.4432↓ |  | 92.3756↓ | 93.925↓ | 127.5260 |
| 62 | Peroxiredoxin 4 | PRDX4 | 3TKS | DN |  | 134.829↑ | 69.9574↓ |  | 88.0082↓ | 113.583↑ | 98.0696 |
| 63 | Rho-associated protein kinase 1 | ROCK1 | 2etk | DN |  | 113.972↑ | 56.0626↓ |  | 80.4755↓ | 82.5391↓ | 98.5533 |
| 64 | soluble epoxide hydrolase | SEH | 1vj5 | DN |  | 120.018↑ | 60.8333↓ |  | 84.5916↓ | 101.876↑ | 96.5568 |
| 65 | TGF-beta receptor type I | TGFR-1 | 1py5 | DN |  | 152.781↑ | 73.8507↓ |  | 101.654↑ | 116.621↑ | 94.2892 |
| 66 | Tyrosine-protein kinase | JAK2 | 2b7a | DN |  | 128.486↑ | 55.081↓ |  | 95.9721↓ | 103.71↓ | 114.0960 |
| 67 | A disintegrin and metalloproteinase with thrombospondin motifs 4 | ADAMTS-5 | 2rjp | MD |  | 121.067↓ | 76.4661↓ |  | 91.4654↓ | 120.413↓ | 190.3480 |
| 68 | Aminopeptidase N | HAPN | 4fyr | MD |  | 97.9778↑ | 48.9276↓ |  | 64.9035↑ | 65.9316↑ | 64.8627 |
| 69 | Amyloid beta A4 protein | ABPP | 5csz | MD | 156.256↓ | 125.307↓ | 54.5627↓ |  | 86.8643↓ | 100.21↓ | 194.4930 |
| 70 | Cathepsin D | CD | 4obz | MD | 145.588↑ | 116.515↑ | 45.6014↓ | 122.268↑ | 71.902↓ | 79.4376↓ | 96.5604 |
| 71 | Copper-transporting ATPase 2 | CT2 | 3cjk | MD | 83.5548↑ | 107.506↑ | 61.5337↓ | 35.8165↓ | 81.9426↑ | 82.8419↑ | 75.4360 |
| 72 | Glycogen synthase kinase-3 beta | GSK-3 beta | 1q3d | MD | 84.9713↓ | 105.215↓ | 51.2015↓ | 111.8↑ | 84.4826↓ | 95.8905↓ | 106.5580 |
| 73 | Hypoxia-inducible factor 1-alpha | HIF-1α | 3hqu | MD | 125.059↓ | 98.6025↓ | 52.1128↓ | 78.5377↓ | 76.7633↓ | 74.4368↓ | 186.0210 |
| 74 | Integrin beta-7 | ITGB7 | 3v4v | MD | 113.335↓ | 102.406↓ | 48.8858↓ | 105.704↓ | 79.925↓ | 79.0646↓ | 128.8320 |
| 75 | Protein DJ-1 | PDJ-1 | 3cza | MD |  | 51.0368↑ |  |  |  |  | 42.6154 |
| 76 | Interleukin-1 receptor antagonist protein | IL1RN | 1IRA | DN，MD | 66.1904↑ | 90.6471↑ | 41.2349↓ | 59.2543↓ | 68.8547↑ | 64.6625↑ | 63.1654 |
| 77 | Malonyl-CoA decarboxylase, mitochondrial | MLYCD | 4f0x | DN，MD |  | 121.958↑ | 71.5558↓ | 126.564↑ | 92.5244↓ | 97.0385↓ | 116.4550 |
| 78 | Mammalian target of Rapamycin | mTOR | 1fap | DN，MD | 134.018↑ | 112.884↓ | 37.0022↓ | 113.064↓ | 84.6974↓ | 81.5902↓ | 129.2840 |
| 79 | Superoxide dismutase [Mn] | SOD2 | 2adp | DN，MD |  | 102.44↑ | 67.9341↓ |  | 84.1427↓ | 73.0139↓ | 74.5191 |

Appendix 2:the result of docking between the molecules and proteins
