# Supplementary figures and images for: Revealing Synergistic Mechanism of Multiple Components in Gandi Capsule for Diabetic Nephropathy Therapeutics by Network Pharmacology
Source: Evid Based Complement Alternat Med. 2018 Apr 26;2018:6503126. doi: 10.1155/2018/6503126 (PMC5944259; doi:10.1155/2018/6503126)

# Graphical Abstract

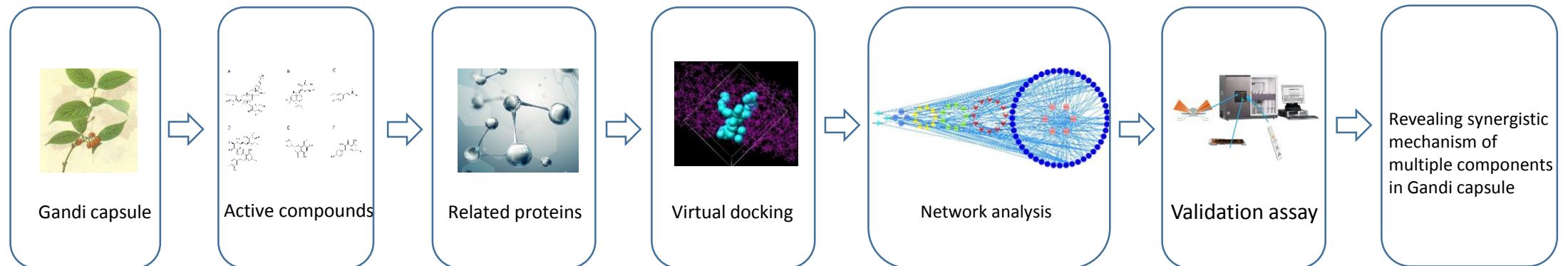

Supplement: Supplementary Materials — Six files have been uploaded as supplementary materials for this research. The first file is the WORD file of the appendix, which has two forms. One is the target protein database, and the other is the form of valid results between the compounds and proteins in docking. The second file is an EXCEL file of the compound database including 315 compounds. The third file is the graphical abstract in PDF, which shows the main process of the research with pictures. The fourth file is a form with three sheets in EXCEL format, which includes total results of docking, valid results of docking, and high affinity results of docking. Meanwhile, the fifth file and the sixth file are EXCEL files of the network diagram of representative target-compound networks and target-compound-pathway networks, respectively. [file 6503126.f1.zip › 6503126.f1/Graphical Abstract.pdf]
